# Clinical study of isokinetic training with joint control tasks on lower limb function in stroke patients with proprioceptive impairment: a preliminary randomized controlled trial

**DOI:** 10.3389/fneur.2026.1881854

**Published:** 2026-10-12

**Authors:** Zhendong Zhang, Huixian Yu, Ping Li, Na Li, Yumei Zhang

**Affiliations:** 1 Department of Neurology, Beijing Tiantan Hospital, Capital Medical University, Beijing, China; 2 Department of Rehabilitation Medicine, Beijing Tiantan Hospital, Capital Medical University, Beijing, China; 3 Beijing Neurosurgical Institute, Beijing Tiantan Hospital, Capital Medical University, Beijing, China

**Keywords:** activities of daily living (ADL), isokinetic training with joint control tasks, motor function, proprioceptive impairment, stroke

## Abstract

**Objective:**

To investigate the effects of isokinetic training with joint control tasks on lower limb proprioceptive and motor function in stroke patients with proprioceptive impairment.

**Methods:**

A randomized controlled trial design was adopted. Stroke patients with proprioceptive dysfunction who were admitted to the Department of Rehabilitation Medicine, Beijing Tiantan Hospital, Capital Medical University, between April 2025 and November 2025 were prospectively and consecutively enrolled. Participants were randomly assigned to an experimental group or a control group using a random number table. Both groups received conventional rehabilitation for 4 weeks, while the experimental group additionally underwent isokinetic training with joint control tasks during the same intervention period. General demographic and clinical data were collected. Before and after intervention, proprioception, motor function, and activities of daily living (ADL) were assessed.

**Results:**

Based on the predefined inclusion and exclusion criteria, 58 eligible patients were finally enrolled and randomly allocated into two groups (29 patients per group). Within-group comparisons showed that both groups exhibited significant post-intervention improvements in proprioceptive function, motor function, and activities of daily living (ADL) (all *P* < 0.05). In the control group, the Modified Nottingham Sensory Assessment (NSA), Joint Position Reproduction (JPR) tests at 30°, 45°, and 60°, Threshold to Detect Passive Motion (TTDPM), Berg Balance Scale (BBS), Fugl–Meyer Assessment for the lower extremity (FMA-LE), and Modified Barthel Index (MBI) scores all improved significantly compared with baseline (*P* < 0.05). Similarly, in the experimental group, all aforementioned indicators demonstrated significant improvements after intervention (*P* < 0.05). Between-group comparisons revealed that improvements in proprioceptive, motor, and ADL functions were significantly greater in the experimental group than in the control group. Specifically, the changes in NSA, JPR (30°, 45°, 60°), and TTDPM scores were significantly higher in the experimental group (*P* < 0.05). Furthermore, the experimental group exhibited larger gains in BBS and FMA-LE scores (*P* < 0.05), as well as greater improvements in MBI scores (*P* < 0.05).

**Conclusion:**

Isokinetic joint control training combined with conventional rehabilitation was associated with improvements in proprioceptive function, lower-limb motor performance, and activities of daily living in stroke patients with proprioceptive impairment. These findings suggest that this approach may serve as a useful adjunct to stroke rehabilitation; however, further studies with more rigorous designs are required to confirm these results.

## 1 Introduction

Proprioception refers to the awareness of joint movement, body motion, and spatial position (1). It provides real-time sensory feedback essential for precise motor control (2). In stroke survivors, the incidence of proprioceptive impairment is as high as 60% (3). Such deficits markedly compromise balance, coordination, and motor control, thereby hindering recovery of independence in daily living (4–7). Despite its clinical relevance, proprioceptive dysfunction has received considerably less attention than motor deficits in both clinical practice and stroke rehabilitation research (8, 9). Compared with the evidence on motor recovery, targeted assessment and therapeutic strategies for proprioceptive dysfunction remain limited, and a standardized clinical framework has yet to be established.

Isokinetic training with joint control tasks, a novel rehabilitation approach, has been applied in several clinical contexts, with promising evidence supporting its role in enhancing proprioceptive feedback. In a randomized controlled trial, Wang et al. (10) demonstrated that such training improved knee strength, proprioception, and balance in athletes following anterior cruciate ligament reconstruction. Similarly, Gezginaslan et al. (11) reported that this training modality effectively enhanced proprioception and balance in patients with knee osteoarthritis, thereby reducing fall risk.

However, current applications of isokinetic training with joint control tasks in stroke rehabilitation primarily focus on improving motor function (12), while its specific effects on proprioceptive recovery remain largely unexplored in neurological populations. Considering its established benefits in orthopedic rehabilitation and its mechanistic potential to enhance sensorimotor integration, applying isokinetic training with joint control tasks to stroke patients with proprioceptive deficits may hold substantial therapeutic promise.

Therefore, this study aimed to investigate the effects of isokinetic training with joint control tasks on proprioceptive and motor function in stroke patients with proprioceptive impairment, with particular emphasis on its impact on lower limb proprioceptive recovery. Given the well-documented body-part specificity of proprioceptive accuracy (13), the present study focused on the lower limb, using knee joint proprioceptive acuity as the primary outcome measure.

## 2 Materials and methods

### 2.1 Ethics

Ethical approval for this study was obtained from the IRB of Beijing Tiantan Hospital, Capital Medical University (No. KY2025-066-02). In accordance with the Declaration of Helsinki, all the subjects were informed about the purpose and procedure of the study and gave their informed consent to participate in the study.

### 2.2 Design and setting

This study prospectively and consecutively enrolled stroke patients with proprioceptive impairment who were admitted to the Department of Rehabilitation Medicine, Beijing Tiantan Hospital, Capital Medical University, between April 2025 and November 2025. The study was designed as a randomized controlled trial (RCT) and was registered in the Chinese Clinical Trial Registry (Registration number: ChiCTR2500111205).

### 2.3 Participants

Both ischemic and hemorrhagic stroke patients were eligible for inclusion. Inclusion criteria: Meeting the diagnostic criteria for stroke established by the Cerebrovascular Disease Group of the Neurology Branch of the Chinese Medical Association in the Key Points for the Diagnosis of Various Major Cerebrovascular Diseases in China (2019) (14), confirmed by cranial CT or MRI; First-ever stroke, aged 18–80 years, with unilateral limb dysfunction; Course of disease between 2 weeks and 6 months; Brunnstrom stage of the affected lower limb ≥ stage III, with muscle strength of knee flexors and extensors ≥ grade 3; Clinical examination confirming proprioceptive impairment (diminished or absent proprioception); Written informed consent obtained from the patient or their legal representative. Exclusion criteria: Severe lower limb spasticity (Modified Ashworth Scale score ≥ 3 in knee flexors or extensors); Deep sensory deficits due to other causes, such as spinal cord injury; Lower limb joint pain due to other conditions (e.g., sprain, acute arthritis, meniscal injury); Cognitive impairment(Montreal cognitive function assessment scale,MoCA) < 20; Unilateral neglect or apraxia; Severe hepatic, renal, or cardiac dysfunction, or malignant hypertension.

### 2.4 Qualification procedure for intervention

Patients who met the inclusion criteria were assessed by a rehabilitation physician. Those who passed the qualification assessment were randomly assigned to one of two groups: (1) the control group, which received conventional rehabilitation therapy; (2) the Experimental group, which received conventional rehabilitation combined with isokinetic training with joint control tasks.

All participants underwent two evaluations: the first assessment was conducted on the day of admission to the rehabilitation center, before the intervention began, and the second was performed after completing the 4-week intervention program.

### 2.5 Rehabilitation program

#### 2.5.1 Conventional rehabilitation

Conventional rehabilitation aimed to promote recovery of motor control and neuroplasticity after stroke through motor relearning and sensory feedback integration (15). The rehabilitation program consisted of motor therapy and proprioceptive training, conducted twice daily, 5 days per week, for four consecutive weeks.

(1) Motor therapy: this included range of motion exercises, muscle stretching, balance and gait training, and strengthening and endurance exercises (16). The underlying mechanism is underpinned by the repetitive, active motor practice, which stimulates neuroplastic reorganization in the cortical motor areas and the corticospinal tract. This reorganization is evidenced by enhanced motor evoked potentials (MEPs), increased cortical excitability, and improved functional connectivity/integration between the cortex and sensorimotor regions, ultimately leading to the restoration of motor control (17, 18). Depending on the patient's functional status, classical facilitation techniques such as Brunnstrom stage training or Bobath neurodevelopmental treatment (NDT) were incorporated. The former facilitates progressive recovery of movement patterns to achieve functional transfer (19), whereas the latter promotes normal movement through postural control and reflex inhibition (20). Additionally, device-assisted training (e.g., upper and lower limb passive/active training systems, gait training systems, or virtual reality-based training) was included to enhance training intensity, repetition, and opportunities for neural network reorganization (16, 21).

(2) Proprioceptive training: this targeted enhancement of sensory pathway activation (muscle spindles, Golgi tendon organs, joint receptors) to improve central perception of body position and movement (22). The main interventions included: Somatosensory stimulation, such as tactile pressure and vibration, to activate the sensory cortex and promote sensorimotor network reorganization (23); Proprioceptive neuromuscular facilitation (PNF), which applies specific stretching and contraction patterns to stimulate proprioceptors and enhance neuromuscular coordination and stability (24).

#### 2.5.2 Isokinetic training with joint control tasks

Isokinetic training was performed using the CON-TREX MJ, Multi-joint Isokinetic Testing and Training System (Tianjin Physioman Health Technology Co., Ltd) (25). Before training, limb weight was compensated by the system to eliminate the influence of gravity. During training, patients were instructed to flex and extend the affected knee joint with maximal effort, adjusting their exertion based on real-time visual feedback on the computer screen. Training intensity was adjusted to ensure the patient experienced no significant fatigue or discomfort on the following day.

(1) Standard position: participants were seated at a 90° angle, holding the chair handles. The trunk and thigh were secured with straps, and the foot was fixed to the lever arm at the ankle level.

(2) Exercise protocol: the training mode was set to “Training – Isokinetic – Parabolic” under the “Concentric/Concentric” mode. The anatomical zero position was defined as 0° of knee flexion, and the range of motion was set between 0° and 90° of flexion. Based on and modified from Gezginaslan et al. (11), the exercise protocol included 10 angular velocities: 60°/s, 90°/s, 120°/s, 150°/s, 180°/s, 180°/s, 150°/s, 120°/s, 90°/s, and 60°/s, with a 30°/s increment between each speed. Each velocity involved 10 repetitions, totaling 100 contractions per session. A 60-s rest interval was provided between velocity sets. Training frequency: once daily, 5 days per week, for 4 weeks.

### 2.6 Data collection

Demographic and clinical data were collected, including: (1) demographic variables: sex, age, etc.; (2) modifiable cerebrovascular risk factors: hypertension, diabetes, smoking history, alcohol consumption; (3) stroke-related information: disease duration, stroke type, and lesion side.

### 2.7 Rehabilitation assessment

#### 2.7.1 Primary outcome

##### 2.7.1.1 Proprioceptive function

(1) Modified Nottingham Sensory Assessment (NSA): The reliability of the NSA for assessing proprioceptive impairment in adult stroke patients has been validated (26). A culturally adapted Chinese version with high reliability and validity was established by Yang et al. in 2021 (27). Assessment procedure: patients lay supine with their head and shoulders supported. The examiner passively moved the patient's limb at a given joint, and the patient verbally described the direction of movement. The four-level scoring system was as follows: 0 = no sensation regardless of movement; 1 = vague sensation with inability to describe the direction; 2 = correct direction identification only for movements >10°; 3 = correct direction identification even for small movements ≤ 10 (27).

(2) Joint Position Reproduction Test (JPR): The JPR test is one of the most widely used methods for assessing proprioceptive acuity (28). Testing was conducted using the CON-TREX MJ, Multi-joint Isokinetic Testing and Training System (Tianjin Physioman Health Technology Co., Ltd, Tianjin, China), with a customized testing protocol according to the study design. Test position: the participant sat with the thigh resting flat on the seat, the popliteal fossa aligned with the seat edge, and the trunk secured with a safety belt. The dynamometer axis was aligned with the lateral femoral condyle of the affected knee, and the resistance pad was fixed 3 cm above the medial malleolus. The unaffected limb hung freely. The range of motion was set with 0° as full knee extension. Test angles were 30°, 45°, and 60° of knee flexion (11). External sensory cues were eliminated using cushions, blindfolds, and earplugs (29). Example (60° JPR): from 0°, the knee was passively flexed to 60° and held for 10 s. The participant memorized the position, then the limb was passively returned to 0° for 10 s. The participant was asked to reproduce the memorized angle. Three trials were performed at 5-min intervals, and the mean absolute error (AE) between the target and reproduced angles was calculated. A smaller AE indicates better position sense.

(3) Threshold to Detection of Passive Motion (TTDPM) (1, 13): as in the JPR test, external cues were blocked. Testing used the same CON-TREX system. Test position: identical to that in the JPR test, with a reference angle of 90° knee flexion. Procedure: the affected knee was passively moved by the dynamometer in a random direction (flexion or extension). The participant was instructed to press a button immediately upon detecting movement and indicate the perceived direction. Incorrect responses were discarded and retested until three correct detections were recorded. The smallest detectable angular displacement from the 90° reference position was measured, and the mean of the three absolute differences was calculated. Smaller mean angles indicated better movement sense.

#### 2.7.2 Secondary outcomes

##### 2.7.2.1 Motor function assessment

(1) Fugl-Meyer Assessment of Lower Extremity (FMA-LE): the FMA-LE is a reliable and valid tool for evaluating motor function of the lower limb (30). It consists of 17 items, each scored on a 3-point ordinal scale (0–2 points), with a total score of 34. Higher scores indicate better motor function.

(2) Berg Balance Scale (BBS): The BBS is considered the gold standard for assessing balance ability and demonstrates good reliability and validity (31). It comprises 14 balance-related tasks, including sitting, standing, and single-leg stance. Each item is rated on a 5-point scale (0–4 points), with a total score of 56. Higher scores represent better balance performance.

##### 2.7.2.2 Activities of daily living (ADL) assessment

The Modified Barthel Index (MBI) was used to evaluate the level of independence in activities of daily living. Compared with the original version, the MBI shows higher reliability and validated psychometric properties (32). The MBI comprises 10 items, each rated on a 5-level scale (Levels 1 to 5). Level 1 denotes complete dependence, and Level 5 signifies complete independence. The total score is 100, with higher scores indicating better activities of daily living (ADL) capacity.

### 2.8 Sample size

The primary outcome of this study was the Joint Position Reproduction Error (JPR Error, unit: degrees, °) of the knee joint. Based on a previous study (33), a moderate effect size (Cohen's *d* ≈ 0.8) was expected in proprioception improvement between groups after intervention. The significance level was set at α = 0.05 (two-tailed), and the statistical power at 1–β = 0.8. Sample size was calculated using the two-sample *t*-test formula: $n=2\times {\left(\frac{Z_{1-\alpha /2}+Z_{1-\beta }}{d}\right)}^{2}$, yielding *n* = 25 per group. Considering an anticipated 10% dropout rate, the adjusted sample size was *n* = 28 per group, totaling 56 participants. In this study, 58 participants were enrolled (29 in each group), meeting the required sample size.

### 2.9 Randomization and allocation

After eligibility was confirmed by a rehabilitation physician and written informed consent was obtained, eligible participants were enrolled in the study. Researcher A generated the randomization sequence using a random number table and was responsible for assigning participants to either the experimental group or the control group according to the generated sequence. Allocation concealment was not implemented because of practical limitations. The outcome assessors were aware of participants' group assignments during the assessment process; therefore, outcome assessment was not blinded. The potential implications of the lack of allocation concealment and assessor blinding are discussed in the Limitations section.

### 2.10 Statistical analysis

All statistical analyses were performed using Stata/MP 18.0 (StataCorp LLC, College Station, TX, USA). Continuous variables were assessed for normality using the Shapiro–Wilk test. Normally distributed continuous variables were expressed as mean ± standard deviation (SD), whereas non-normally distributed continuous variables were presented as median and interquartile range (IQR). Categorical variables were expressed as frequencies and percentages.

Within-group comparisons were performed separately for the experimental and control groups to evaluate changes from baseline to post-intervention. For normally distributed continuous variables, the paired-samples *t*-test was used, whereas the Wilcoxon signed-rank test was used for non-normally distributed continuous variables.

Between-group comparisons were performed to evaluate differences in treatment effects between the experimental and control groups. For this purpose, the change score for each outcome was calculated as the post-intervention value minus the baseline value. The change scores were then compared between the two groups using the independent-samples *t*-test for normally distributed data or the Mann–Whitney *U*-test for non-normally distributed data.

Categorical variables were compared using the chi-square test or Fisher's exact test, as appropriate. All statistical tests were two-sided, and *P* < 0.05 was considered statistically significant.

The joint position reproduction error (JPR error) was considered the primary outcome of this preliminary randomized controlled trial, while the other clinical measures were considered secondary or supportive outcomes. Within-group comparisons were primarily used to characterize changes from baseline to post-intervention, whereas between-group comparisons of change scores were used to evaluate differences in treatment effects between the two groups. Given the preliminary nature of this study and the distinction between the primary outcome and secondary/supportive outcomes, no global multiplicity adjustment was applied to all statistical comparisons. Therefore, the reported *P* values should be interpreted in conjunction with the corresponding effect sizes and 95% confidence intervals, and findings from secondary or supportive analyses should be interpreted cautiously.

Effect sizes (Cohen's *d*) were interpreted according to the classification criteria proposed by Cohen (34): 0.2–0.49 indicates a small effect, 0.5–0.79 a medium effect, and ≥0.8 a large effect. Effect sizes (correlation coefficient *r*) were interpreted based on the classification criteria from Cohen (34) and Fritz et al. (35): 0.10–0.29 denotes a small effect, 0.30–0.49 a medium effect, and ≥0.50 a large effect.

## 3 Results

### 3.1 Participant characteristics and baseline comparability

A total of 58 patients were included in the analysis (29 in each group) after screening based on inclusion and exclusion criteria. No adverse events occurred during the study. Table 1 presents the baseline demographic and clinical characteristics. No significant differences were found between the two groups in age, sex, stroke type, disease duration, lesion side, or comorbidities such as hypertension and diabetes (*P* > 0.05). Table 2 shows baseline comparisons of the primary and secondary outcome measures, including proprioceptive function, motor function, and ADL ability. No significant between-group differences were observed (*P* > 0.05), indicating good group comparability following randomization.

**Table 1:** Baseline demographic and clinical characteristics of participants.

Demographic Characteristics	Control (*n* = 29)	Experimental (*n* = 29)	Statistical analysis (*t*/*U*/χ^2^)	*P*-value
Age (years)	59.76 ± 12.49	56.93 ± 13.28	0.836	0.407
Gender (males/females)	20/9	21/8	0.083	0.773
Type of stroke (ischemic/hemorrhagic)	22/7	21/8	0.090	0.764
Time post stroke (days)	16 (14, 25)	17 (14, 25)	−0.437	0.662
Lesion side (right/left)	12/17	17/12	1.724	0.189
Hand Dominance (right/left)	29/0	28/1	1.018	0.313
Smoking history (yes/no)	13/16	17/12	1.105	0.293
Drinking history (yes/no)	10/19	17/12	3.400	0.065
Hypertension (yes/no)	19/10	18/11	0.075	0.785
Diabetes mellitus (yes/no)	9/20	10/19	0.078	0.780

**Table 2:** Baseline comparisons of primary and secondary outcome measures.

Outcome measures	Control (*n* = 29)	Experimental (*n* = 29)	Statistical analysis (*t*/*U*)	*P*-value
NSA	2 (2, 3)	2 (2, 2)	1.900	0.064
JPR_30° (°)	4.4 (2.73, 5.73)	4.5 (3.93, 7.17)	−1.735	0.084
JPR_45° (°)	4.07 (2.6, 5.57)	3.97 (3.27, 5.57)	−0.242	0.814
JPR_60° (°)	3.43 (2.77, 4.86)	4.5 (3.29, 6.03)	−1.828	0.068
TTDPM (°)	2.97 (1.57, 5.1)	4.3 (2.6, 6.5)	−1.488	0.139
BBS	31.48 ± 13.17	29 ± 12.40	0.713	0.479
FMA-LE	21.37 ± 6.36	22.22 ± 5.38	−0.531	0.598
MBI	63.93 ± 17.78	61.33 ± 18.16	0.53	0.598

NSA, modified nottingham sensory assessment; JPR_30°, joint position reproduction test at 30 degrees; JPR_45°, joint position reproduction test at 45 degrees; JPR_60°, joint position reproduction test at 60 degrees; TTDPM, threshold to detection of passive motion; BBS, berg balance scale; FMA-LE, Fugl-Meyer assessment of lower extremity; MBI, modified barthel index.

### 3.2 Effects on lower-limb proprioception

As shown in Tables 3, 4, and Figures 1, 2, both groups demonstrated significant improvements in lower-limb proprioception after the 4-week intervention. Figures 1, 2 present the distributions of individual participant measurements at baseline and after intervention, together with the changes observed within each group. The individual-level data show an overall shift toward improved proprioceptive performance after treatment, while also illustrating the variability of responses among participants.

**Figure 1:**
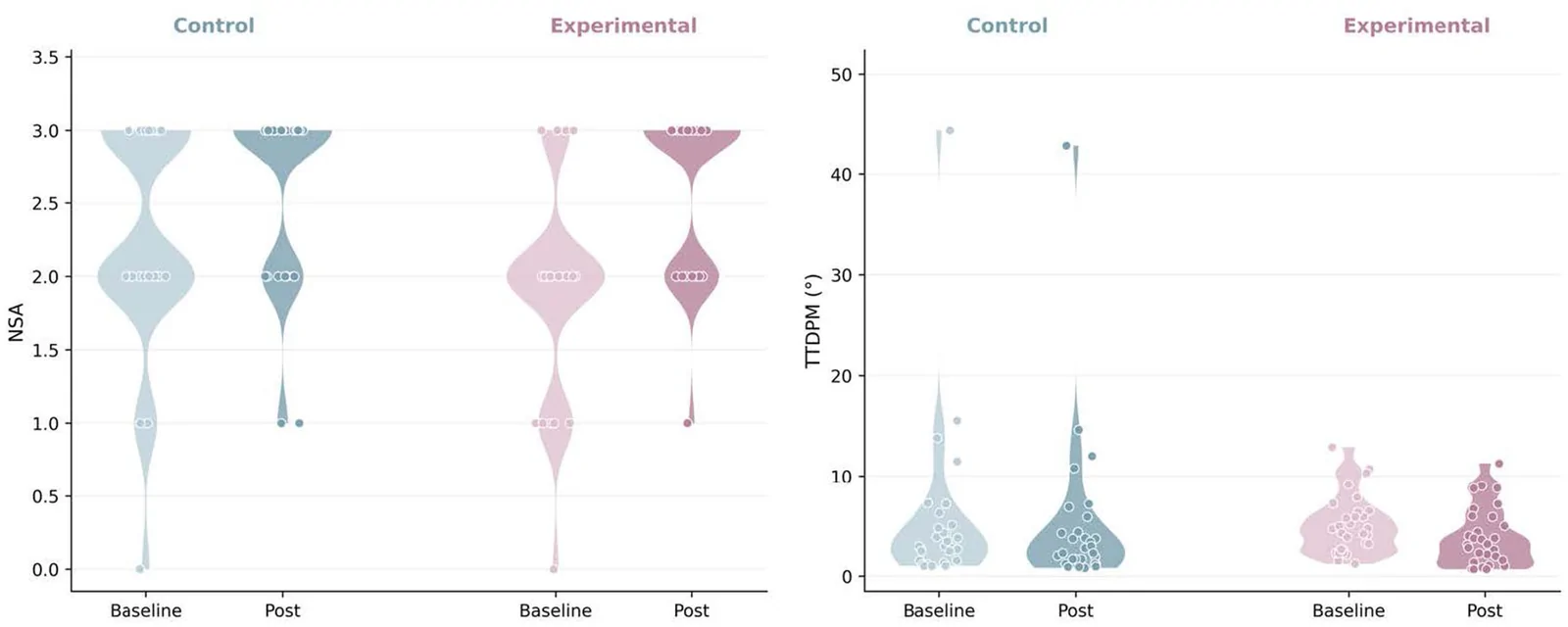
Changes in lower-limb proprioception.

**Figure 2:**
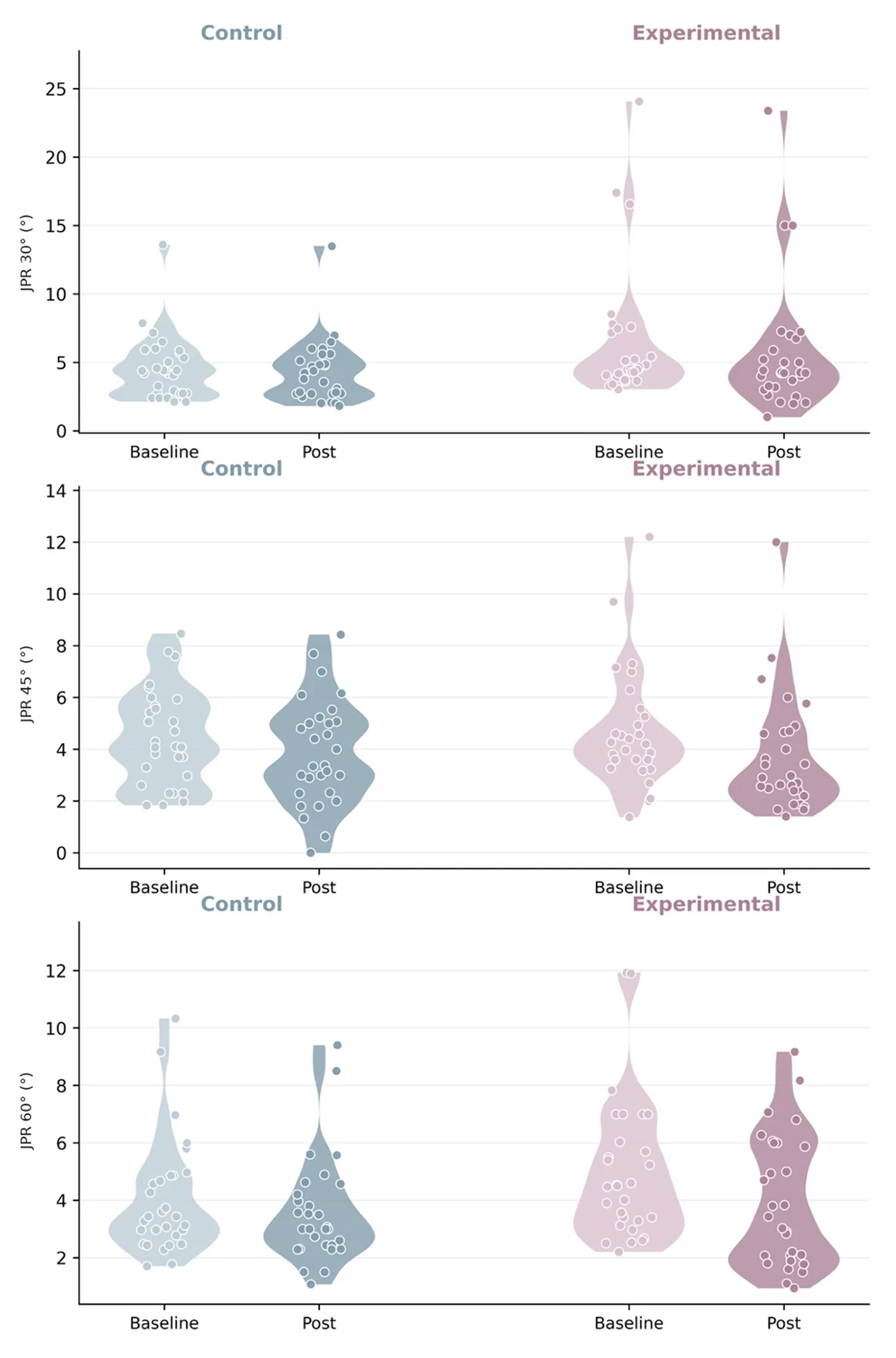
Changes in joint position reproduction error.

**Table 3:** Within-group comparisons of outcome changes in the control group (*n* = 29).

Outcome measures	Change-value	*P*-value	Statistical analysis (*t*/*U*)	Cohen's *d*/*r*	95%CI
NSA	0.34 ± 0.61	0.005	3.025	0.562	0.11, 0.58
JPR_30° (°)	−0.25 ± 0.48	0.010	−2.765	−0.513	−0.43, −0.06
JPR_45° (°)	−0.36 (−0.97, −0.07)	0.001	−4.239	0.787	−0.72, −0.22
JPR_60° (°)	−0.45 ± 0.40	0.001	−6.058	−1.125	−0.61, −0.30
TTDPM (°)	−0.5 (−0.67, −0.2)	< 0.001	−3.364	0.625	−0.59, −0.33
BBS	12.69 ± 8.95	0.001	7.636	1.418	9.29, 16.09
FMA-LE	5.69 ± 3.68	0.001	8.337	1.548	4.29, 7.09
MBI	14 (4, 19)	< 0.001	4.431	0.823	8.10, 16.56

**Table 4:** Within-group comparisons of outcome changes in the experimental group (*n* = 29).

Outcome measures	Change-value	*P*-value	Statistical analysis (*t*/*U*)	Cohen's *d*/*r*	95%CI
NSA	0.69 ± 0.60	< 0.001	6.151	1.142	0.46, 0.92
JPR_30° (°)	−0.81 ± 0.87	< 0.001	−4.982	−0.925	−1.14, −0.48
JPR_45° (°)	−0.94 (−1.7, −0.37)	0.001	−4.076	0.757	−1.38, −0.62
JPR_60° (°)	−1.07 ± 1.42	< 0.001	−4.033	−0.749	−1.61, −0.52
TTDPM (°)	−1.2 (−1.6, −0.67)	0.001	−4.617	0.857	−1.42, −0.90
BBS	18.10 ± 8.95	0.001	10.888	2.022	14.70, 21.51
FMA-LE	7.90 ± 3.44	0.001	12.374	2.298	6.59, 9.20
MBI	22 (12, 30)	< 0.001	3.966	0.736	16.29, 26.37

In the control group, significant within-group improvements were observed in NSA, JPR at 30°, 45°, and 60°, and TTDPM (all *P* < 0.05), with effect sizes ranging from moderate to large (Cohen's *d* = 0.513–1.125; *r* = 0.625–0.787). In the experimental group, significant improvements were also observed across all proprioceptive measures (all *P* < 0.05), with effect sizes ranging from moderate to large (Cohen's *d* = 0.749–1.142; *r* = 0.757–0.857).

Between-group comparisons of change scores further demonstrated significantly greater improvements in the experimental group for all proprioceptive outcomes (all *P* < 0.05; Table 5). NSA, JPR (30°, 45°, 60°), and TTDPM all improved significantly more in the experimental group, with small to moderate effect sizes (e.g., NSA: Cohen's *d* = −0.566; TTDPM: *r* = 0.451).

**Table 5:** Between-group comparisons of change scores in primary and secondary outcomes.

Outcome measures	Control (*n* = 29) change-value	Experimental (*n* = 29) change-value	*P*-value	Statistical analysis (*t*/*U*)	Cohen's *d*/*r*	95%CI
NSA	0.34 ± 0.61	0.69 ± 0.60	0.035	−2.157	−0.566	−0.67, −0.02
JPR_30° (°)	−0.25 ± 0.48	−0.81 ± 0.87	0.004	3.033	0.643	0.19, 0.93
JPR_45° (°)	−0.36 (−0.97, −0.07)	−0.94 (−1.7, −0.37)	0.033	2.131	0.280	−0.97, −0.10
JPR_60° (°)	−0.45 ± 0.40	−1.07 ± 1.42	0.033	2.232	0.431	0.05, 1.17
TTDPM (°)	−0.5 (-−0.67, −0.2)	−1.2 (−1.6, −0.67)	< 0.001	3.438	0.451	−0.98, −0.42
BBS	12.69 ± 8.95	18.10 ± 8.95	0.025	−2.303	−0.605	−10.12, −0.70
FMA-LE	5.69 ± 3.68	7.90 ± 3.44	0.022	−2.362	−0.620	−4.08, −0.34
MBI	14 (4, 19)	22 (12, 30)	0.010	−2.572	0.338	4.33, 13.67

### 3.3 Effects on lower-limb motor and balance function

Tables 3, 4, and Figure 3 summarize the changes in lower-limb motor and balance function following the intervention. Both groups showed significant improvements in FMA-LE and BBS scores after treatment, indicating improvements in lower-limb motor function and balance performance.

**Figure 3:**
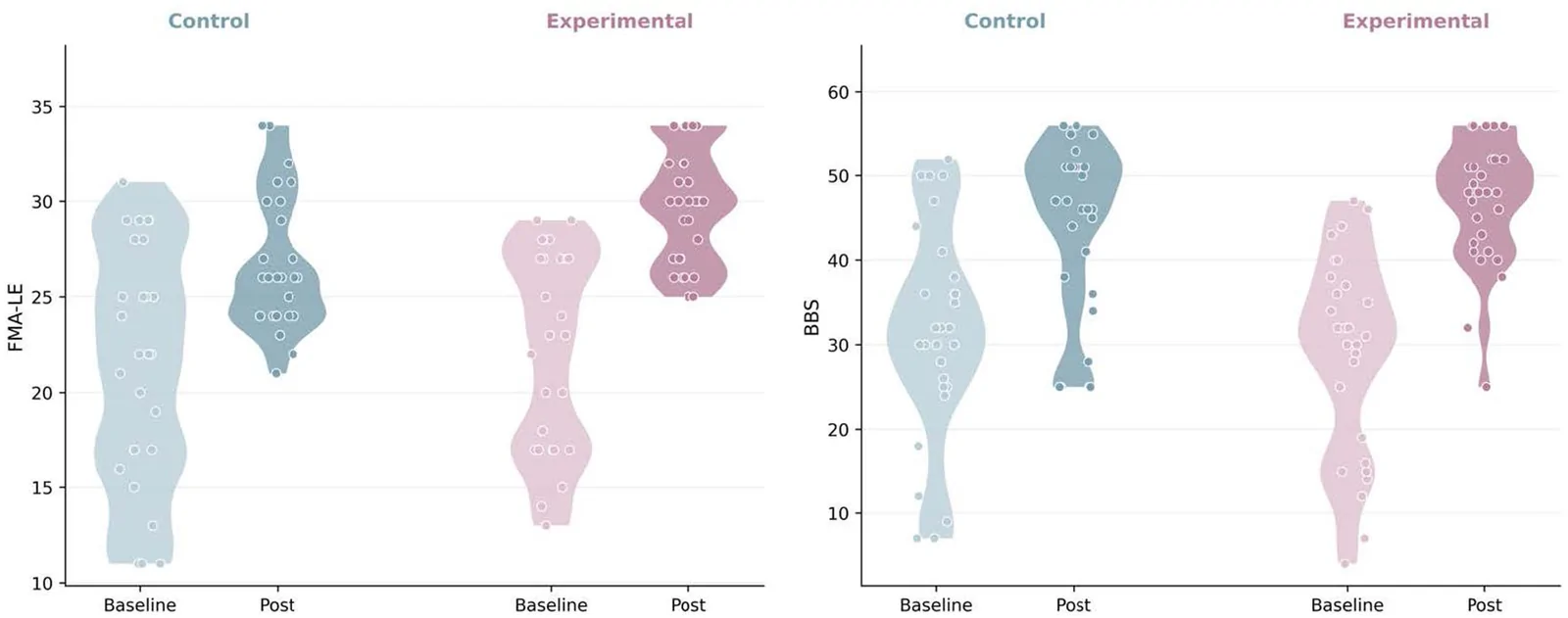
Changes in lower-limb motor and balance function.

In the control group, FMA-LE and BBS increased by 5.69 ± 3.68 and 12.69 ± 8.95 points, respectively, with large within-group effect sizes (*d* = 1.548 and 1.418, respectively). In the experimental group, the corresponding increases were 7.90 ± 3.44 points for FMA-LE and 18.10 ± 8.95 points for BBS, with larger effect sizes (*d* = 2.298 and 2.022, respectively). Between-group comparisons showed significantly greater improvements in the experimental group for both FMA-LE and BBS (all *P* < 0.05), with between-group effect sizes of 0.620 and 0.605, respectively.

Figure 3 illustrates the baseline-to-post-intervention distributions of FMA-LE and BBS scores in both groups. Overall, the figure demonstrates greater upward shifts in motor and balance function in the experimental group, while individual data points show the distribution and variability of treatment responses.

### 3.4 Effects on activities of daily living

As shown in Tables 3–5, activities of daily living also improved significantly after the intervention in both groups. The MBI increased by 14 (4, 19) points in the control group and 22 (12, 30) points in the experimental group, with large within-group effect sizes (*r* = 0.823 and 0.736, respectively). The between-group comparison showed a significantly greater improvement in the experimental group (*P* < 0.05; *r* = 0.338, moderate effect).

Overall, the results indicate that both conventional rehabilitation and the combined intervention were associated with improvements in proprioception, lower-limb motor function, balance, and activities of daily living. Compared with conventional rehabilitation alone, the addition of isokinetic training with joint control tasks produced greater improvements in the proprioceptive measures, FMA-LE, BBS, and MBI.

## 4 Discussion

This randomized controlled trial investigated the effects of isokinetic training with joint control tasks on lower-limb proprioception, motor function, and activities of daily living (ADL) in stroke patients with proprioceptive impairment. The results showed that the addition of isokinetic training to conventional rehabilitation was associated with improvements in proprioceptive function, motor performance, and ADL ability.

Effect size analysis indicated small-to-moderate effects, suggesting that although the intervention did not produce large effects, it still provided clinically meaningful improvements in functional recovery.

### 4.1 Effects of Isokinetic training with joint control tasks on Lower-Limb Proprioception

The results demonstrated that conventional rehabilitation alone could improve proprioceptive function in stroke patients with proprioceptive deficits, supporting the notion that proprioception is plastic and responsive to targeted rehabilitation. Previous studies have confirmed that conventional rehabilitation approaches—such as proprioceptive neuromuscular facilitation (PNF), Bobath therapy, and postural control training—can stimulate proprioceptive receptors through specific manual and sensory techniques. These methods enhance the brain's capacity for functional reorganization by providing tactile and verbal cues for optimal postural control, thereby partially restoring proprioceptive function after stroke (36–38).

In our between-group comparison, isokinetic training with joint control tasks was observed to be associated with improvements in lower-limb proprioception in the post-stroke population. Although the effect size analysis suggested a small-to-moderate effect, the intervention still holds clinical relevance for proprioceptive recovery. Notably, to date, no published randomized controlled trials have directly evaluated the effects of isokinetic training with joint control tasks on proprioception in stroke populations, highlighting the novelty and exploratory value of this study.

While evidence in stroke patients remains limited, multiple RCTs in orthopedic populations (e.g., knee osteoarthritis, anterior cruciate ligament reconstruction) have demonstrated that isokinetic training can enhance joint position sense and kinesthesia (10, 11). Methenitis et al. (39) also reported that both eccentric and concentric modes of isokinetic training improved joint position sense in healthy young men. Collectively, these studies provide an empirical basis for the potential value of isokinetic training with joint control tasks in improving proprioception.

The proprioceptive pathway conveys information about body position, movement, and mechanical forces to the brain for precise motor control and perception (40). Peripheral proprioceptive receptors—such as muscle spindles, Golgi tendon organs, Pacinian corpuscles, and Ruffini corpuscles—play a critical role in this process. The isokinetic training protocol used in this study employed ten different angular velocities, generating repetitive, high-frequency, and controlled stretch–contraction cycles. This training pattern may repeatedly stimulate peripheral proprioceptive receptors and increase proprioceptive afferent input, potentially contributing to improved sensory signal transmission and proprioceptive performance (13). However, these proposed physiological effects were not directly measured in the present study and should therefore be interpreted as possible mechanisms rather than confirmed neurophysiological changes.

A systematic review and network meta-analysis by Zheng et al. (21) demonstrated that proprioceptive training combined with closed kinetic chain exercises (CKCE) or visual feedback training can enhance sensory input and improve balance and gait performance in stroke patients. Isokinetic training with joint control tasks belongs to the CKCE category, as it involves repetitive closed-chain knee movements that stimulate position and motion sense, facilitating neural control of the lower limb—consistent with the findings of Radwan et al. (41) Moreover, visual feedback has been shown to facilitate proprioceptive recovery post-stroke, particularly when sensory training integrates visual cues (42). A multicenter RCT reported that proprioceptive training with visual feedback was superior to non-specific sensory stimulation in improving joint position sense and functional movement of the upper limb (43). In our study, participants could observe real-time torque curves on a visual display during isokinetic training, allowing for quantitative monitoring and feedback. This combination of visual feedback and proprioceptive information may have facilitated sensorimotor integration and contributed to improvements in postural stability and sensory accuracy during functional tasks (21). However, because central sensory integration was not directly assessed in this study, this interpretation remains hypothetical and requires confirmation using objective neurophysiological or neuroimaging measures.

### 4.2 Effects of Isokinetic training with joint control tasks on lower-limb motor function

This study suggested that isokinetic training with joint control tasks was associated with ameliorations in lower-limb motor function among stroke patients with proprioceptive impairment. Both groups showed significant improvements in BBS and FMA-LE scores after intervention, but the experimental group achieved greater gains, indicating an additive effect of isokinetic training. Between-group effect size analysis revealed moderate effects, suggesting clinically meaningful motor improvements.

Mishra et al. (44) reported that proprioceptive training (e.g., weight shifting, single-leg stance) enhances lower-limb balance by repeatedly stimulating proprioceptors and cutaneous mechanoreceptors, increasing the frequency and reliability of sensory input to the central nervous system. In addition to having a similar proprioceptor stimulation pattern, isokinetic training with joint control tasks offer the advantages of adjustable and precise motion speed control mode, as well as controllable and high-frequency “concentric or eccentric” muscle exercise prescriptions. Specifically, this training method can accurately stimulate peripheral proprioceptors, thereby improving the quality of peripheral proprioceptive input — which may be one of the mechanisms underlying the significant improvement in motor function scores observed in this study.

Recent RCTs have shown that combining visual feedback with sensory or balance training can further improve balance and gait in stroke survivors. For example, Yeo et al. (45) found that unstable surface training with visual feedback enhanced BBS scores and gait speed more than conventional training; similarly, Martino Cinnera et al. (46) reported that game-based balance feedback training improved postural stability. In the present study, the CON-TREX multi-joint isokinetic system provided real-time visual feedback via torque curves, likely contributing to superior motor outcomes in the experimental group. The combination of proprioceptive input and visual feedback may also have increased patients' engagement with the training tasks and supported active motor learning. Such repeated sensory–motor practice may potentially contribute to adaptive neural changes after stroke (47); however, neuroplasticity and structural brain changes were not directly assessed in the present study, and this proposed mechanism should therefore be considered hypothetical.

### 4.3 Effects of Isokinetic training with joint control tasks on Activities of daily living

ADL performance was evaluated using the Modified Barthel Index (MBI). Both groups showed improvements after the intervention, with the experimental group exhibiting greater gains. The greater gains observed in the experimental group were associated with the use of isokinetic training with joint control tasks, which was linked to better functional independence. Effect size analysis revealed moderate effects, underscoring its clinical importance.

The observed improvements may be attributed to enhanced joint position sense, movement coordination, and lower-limb stability, which collectively facilitate more efficient performance of standing, walking, and transfer activities (21). Similar findings were reported by Kerimov et al. (12), who demonstrated that isokinetic training improved both motor and functional performance of the paretic upper limb, suggesting that such training not only strengthens muscle performance but also enhances motor coordination and overall functional ability. Furthermore, the visual feedback component increases patient motivation and engagement, indirectly supporting ADL recovery.

Overall, this study suggests that isokinetic training with joint control tasks was associated with improvements in proprioception, lower-limb motor performance, and ADL among stroke patients with proprioceptive deficits. By providing controlled resistance and velocity, this approach appears to offer a safe, precise, and quantifiable training environment that may enhance sensory input quality, muscle coordination, and strength output. Additionally, real-time feedback and closed-chain movement patterns seem to promote active participation and motor relearning, which may facilitate integrated sensory–motor recovery.

## 5 Limitations and future directions

Several limitations should be acknowledged. First, this single-center study had a relatively small sample size, which may limit generalizability. Second, allocation concealment was not implemented, and outcome assessors were not blinded to group assignments. These methodological limitations may have introduced selection bias and assessment bias, respectively, and may have influenced the observed treatment effects. Therefore, the findings should be interpreted with caution. Third, the short intervention period precluded assessment of long-term effects. Fourth, change score analysis, while clinically interpretable, may not fully account for baseline differences; more robust approaches such as ANCOVA could provide better adjustment. Fifth, only clinical scales were used, without neurophysiological or neuroimaging measures to directly investigate the neural mechanisms underlying proprioceptive recovery. Therefore, the proposed explanations involving changes in proprioceptive afferent input, sensorimotor integration, motor learning, and neuroplasticity remain hypothetical and should be interpreted cautiously. Future studies incorporating neurophysiological and neuroimaging assessments are needed to further elucidate the mechanisms underlying the observed clinical improvements. Finally, multiple statistical comparisons were performed without a global adjustment for multiplicity, which may have increased the risk of type I error. Therefore, the findings from secondary and supportive outcomes should be interpreted cautiously and require confirmation in larger, adequately powered randomized controlled trials with prespecified multiplicity strategies.

Given these limitations, the present findings should be interpreted with caution. Nevertheless, we provide preliminary evidence suggesting that isokinetic training with joint control tasks may be associated with improvements in proprioception, motor function, and activities of daily living in stroke patients with proprioceptive impairment. Future multicenter, large-sample RCTs with rigorous allocation concealment, blinded assessment, and objective neurophysiological measures are warranted to confirm these findings.

## 6 Conclusion

Isokinetic training with joint control tasks combined with conventional rehabilitation was associated with significant improvements in proprioceptive function, lower-limb motor performance, and activities of daily living in stroke patients with proprioceptive impairment. By enabling precise control of resistance and movement velocity, this approach may enhance the quality of sensory input and neuromuscular coordination, thereby facilitating sensory-motor integration and functional recovery.

These findings suggest that isokinetic training with joint control tasks may serve as a useful adjunct to conventional stroke rehabilitation. However, the results should be interpreted with caution due to methodological limitations, and further studies with more rigorous designs are needed to confirm these findings.

## Data Availability

The original contributions presented in the study are included in the article/supplementary material, further inquiries can be directed to the corresponding author.
